# Prior Knowledge of Object Associations Shapes Attentional Templates and Information Acquisition

**DOI:** 10.3389/fpsyg.2017.00843

**Published:** 2017-05-23

**Authors:** Rachel Wu, Jiaying Zhao

**Affiliations:** ^1^Department of Psychology, University of California, Riverside, RiversideCA, United States; ^2^Department of Psychology and Institute for Resources, Environment and Sustainability, University of British Columbia, VancouverBC, Canada

**Keywords:** attentional selection, visual search, prior knowledge, statistical learning, categorization

## Abstract

Studies on attentional selection typically use unpredictable and meaningless stimuli, such as simple shapes and oriented lines. The assumption is that using these stimuli minimizes effects due to learning or prior knowledge, such that the task performance indexes a “pure” measure of the underlying cognitive ability. However, prior knowledge of the test stimuli and related stimuli acquired before or during the task impacts performance in meaningful ways. This mini review focuses on prior knowledge of object associations, because it is an important, yet often ignored, aspect of attentional selection. We first briefly review recent studies demonstrating that how objects are selected during visual search depends on the participant’s prior experience with other objects associated with the target. These effects appear with both task-relevant and task-irrelevant knowledge. We then review how existing object associations may influence subsequent learning of new information, which is both a driver and a consequence of selection processes. These insights highlight the importance of one aspect of prior knowledge for attentional selection and information acquisition. We briefly discuss how this work with young adults may inform other age groups throughout the lifespan, as learners gradually increase their prior knowledge. Importantly, these insights have implications for developing more accurate measurements of cognitive abilities.

## Introduction

Studies on attentional selection have typically used unpredictable and meaningless stimuli, such as simple shapes and oriented lines. Even if simple and somewhat meaningful stimuli, such as letters and numerals, are used in a task, different features of the stimuli like color and shape are randomized from trial to trial. The aim of using these unpredictable and minimally meaningful stimuli is to reduce effects due to learning or prior knowledge of the stimuli to obtain a “pure" measure of the underlying cognitive abilities (see Lupyan and Spivey, 2008; Brady et al., 2016). These studies have been instrumental in identifying critical aspects of attentional selection, including the timing and the process of attentional selection. Performance on these tasks, as well as other tasks measuring other cognitive abilities such as working memory, executive function, and inhibition, has been used to determine the range for healthy cognitive development and aging (see Park and Reuter-Lorenz, 2009). These tasks are also used to detect cognitive impairments in aging adults (e.g., Possin et al., 2013). Moreover, cognitive training programs have been using these modularized tasks to improve specific cognitive abilities that they were designed to measure (e.g., Ball et al., 2002; Olesen et al., 2004; Jaeggi et al., 2008).

However, it is difficult, if not impossible, to measure pure cognitive abilities in a single task, because the measurement depends on the participant’s experience with the stimuli and related stimuli prior to the experiment, as well as during the task. Stimuli that are meaningless to the experimenter may not actually be meaningless to the participant, and unpredictable stimuli may disrupt the participant’s existing cognitive models of a generally predictable environment (e.g., Orhan and Jacobs, 2014). In order to obtain more accurate and ecologically valid measures of cognitive abilities, it is important to investigate the influence of prior knowledge, which impacts performance on cognitive tasks in meaningful ways (e.g., Lupyan, 2008; Lupyan and Spivey, 2008; Orhan and Jacobs, 2014; Brady et al., 2016).

This mini review focuses on prior knowledge of associations between individual objects, because it is an important, yet often ignored, aspect of attentional selection. Objects in the natural environment rarely appear on their own. Instead, they almost always appear with other objects. Therefore, it is important to understand how these associations influence attentional selection and subsequent cognitive processes, namely information acquisition. This paper first reviews recent studies showing that the participant’s prior knowledge of objects associated with the search target can either facilitate or hinder search, due to the use and construction of attentional templates. Then, we review studies showing how prior knowledge of associations determines what and how new information is learned, which results from intermediary selection processes. We end with a brief discussion on how this work can inform research with other age groups throughout the lifespan, and aid in developing more accurate measurements of cognitive abilities.

## Prior Knowledge of Task-Relevant Object Associations Shapes Attentional Templates During Visual Search

Recent studies have shown that prior knowledge of object associations shapes how people search for information in the environment. Top-down, or goal-directed, search has been theorized to unfold in the following manner: The participant creates an attentional template, a prioritized working memory representation, of the to-be-searched target item, and then matches the attentional template to the stimuli presented (e.g., Olivers et al., 2011). Without an attentional template, top-down search is inefficient, perhaps impossible. Attentional templates can contain a single feature, a combination of features, a rule, or even a category (Luck and Hillyard, 1994; Eimer, 1996; Nako et al., 2014a; Wu et al., 2016). In many studies investigating attentional selection, the stimuli are simple objects, rather than complex naturalistic objects, in order to minimize the interference of prior knowledge with the visual search process, and to allow an investigation of the “pure” attentional mechanisms and parameters of attentional selection (e.g., Treisman, 1982; Wolfe, 1998; Woodman and Luck, 1999).

Building on the theoretical foundations of top-down search using simple meaningless stimuli, several recent behavioral studies have demonstrated that prior knowledge of object associations indeed impacts attentional templates and search efficiency (Yang and Zelinsky, 2009; Wolfe et al., 2011). For example, during visual search, participants could recall and recognize objects associated with the target more accurately than unrelated distractors (Moores et al., 2003). Distractors in the same color as the target in the natural environment slowed visual search for the target in the laboratory setting, even if the target was grayscale (Olivers et al., 2011). After knowing the target (e.g., banana), participants were slower to orient toward semantically related objects (e.g., monkey) compared to visually related objects (e.g., canoe), demonstrating that semantic biases can be a distraction when task-irrelevant (De Groot et al., 2016). In essence, prior knowledge has benefits and costs on visual search.

Recent ERP studies using the N2pc component suggest that these behavioral benefits and costs may be due to grouping of associated objects into one unit (e.g., a category; Nako et al., 2014a; Wu et al., 2016). When controlling for factors such as salience, the N2pc ERP reflects the number of attentional templates used during a search task (Nako et al., 2014a). Therefore, the N2pc is a useful tool for investigating the grouping of associated objects into an attentional template. In Nako et al. (2014a), participants searched for a letter target among three simultaneously presented distractors from a number category (and vice versa). ERP results revealed that such category search produced similar N2pc components compared to searching for a specific letter target among distractors from the same letter category. In other words, searching for associated objects in one category is similar to searching for a specific object. This finding has been replicated with naturalistic and artificial categories, such as clothing and kitchen items, human and ape faces (Wu et al., 2015), and newly learned Chinese characters and alien families (Wu et al., 2013, 2016). Prior knowledge of object associations also induces costs when distractors are thought to be in the same category as the target or semantically related to the target (Telling et al., 2009; Nako et al., 2014b). For example, when asked to search for the letter “A”, but the letter “R” which is a distractor from the same category as the target appears instead, the participant tends to select the distractor prior to indicating that the target is absent. In these cases, prior knowledge encourages false alarms to distractors related to the target, resulting in poorer behavioral performance when indicating the absence of the target.

These visual search studies dovetail with an increasing body of research on working memory capacity showing that prior knowledge of object and feature associations allows grouping or “compression” of information to overcome memory capacity limitations (e.g., Brady and Alvarez, 2009; Orhan and Jacobs, 2014; Brady et al., 2016; Zhao and Yu, 2016). Costs of prior knowledge emerge when experimental conditions deviate from the statistics in the familiar environment in which the knowledge was first acquired (Green et al., 2010; Orhan and Jacobs, 2014; Blanco et al., 2016). For example, Orhan and Jacobs (2014) argue that using unpredictable stimuli, such as shapes that do not predict color, may induce a “model mismatch” between the current stimuli and the participant’s prior experience in the natural environment, where objects and features are often predictive (e.g., bananas tend to be yellow). This mismatch may negatively impact the participant’s responses when completing the task. Relying on prior knowledge allows people to be more efficient in familiar environments, at the cost of being less efficient in novel environments that encompass different constraints.

## Prior Knowledge of Object Associations Shapes the Construction of Attentional Templates

Prior knowledge induces costs and benefits on attentional selection because it dictates what is included in search templates. This tradeoff due to prior knowledge is the focus of some recent studies investigating how these costs and benefits emerge with learning and experience. The vast majority of visual search studies provide participants with explicit instructions about the target and sometimes the distractors, and assume that the participant creates an attentional template identical to the target shown, or at least containing the relevant features. However, with more complex stimuli containing many features and more ambiguous instructions, what is considered “relevant” can depend heavily on prior and newly acquired knowledge. This notion is consequential because the construction and use of relevant information for attentional templates typically determine search performance, and everyday activities do not often include simple meaningless stimuli or explicit instructions for every action.

Recent studies suggest that the amount of knowledge about object associations acquired prior to and during a task can determine how attentional templates are constructed. For example, newly acquired categories may be more difficult to find initially, but they elicit fewer false alarms compared to highly familiar categories, such as letters and numerals (Wu et al., 2013, 2016). Unfamiliar categories require learning to construct appropriate attentional templates, which may require learning new rules that may be based on seemingly arbitrary principles (e.g., Chinese characters for numbers, Wu et al., 2013). Therefore, search for newly learned categories may be initially inefficient. As the observer becomes more familiar with the categories, false alarms to the non-target items from the target’s category may increase (Wu et al., 2017a). These studies also showed that probabilistic information of object associations can be used to determine which features and objects to prioritize in the attentional template. Such information includes the likelihood of the co-occurrence of objects in a category, which can be used to “chunk” many objects into a unified template (Wu et al., 2013, 2016). For example, Wu et al. (2013) presented English-speaking participants with pairs of objects that belonged in the same category (i.e., Chinese characters for numbers vs. non-numbers), but were not explicitly told what the categories were. Participants implicitly extracted the category information based on the co-occurrence of the characters and formed a unified search template for the two categories of Chinese characters, albeit weaker templates than for familiar letters and numerals. These studies highlight that attentional templates are dynamic, task-specific, and dependent on prior knowledge, perhaps even with “minimally meaningful” stimuli such as letters (see also Nako et al., 2015).

## Prior Knowledge of Task-Irrelevant Object Associations Impacts Search

Prior knowledge of object associations guides the spatial allocation of attention, even when completely task-irrelevant (e.g., Chun and Jiang, 1998; Zhao et al., 2013). In one study (Zhao et al., 2013), “meaningless” abstract novel symbols appeared one after another in a fixed, predictable order (i.e., with regularities), whereas other symbols appeared in a random order. While viewing these “task-irrelevant” objects, participants performed a visual search task where a target (i.e., the letter “T”) appeared in either a structured location where objects appeared in a predictable order or a random location where objects appeared in a random order. Participants were faster to detect the target when it appeared in the structured location compared to the random location, suggesting that attention was biased toward the regularities of the object associations in the structured location. This attentional bias persists even when the regularities are later removed, or when new regularities emerge in a different location (Yu and Zhao, 2015). Moreover, depending on how objects co-occur in space, local and global regularities draw attention to local and global levels, respectively (Zhao and Luo, 2017). These studies demonstrate that the prior knowledge of object associations and co-occurrences biases attention to the spatial location containing regularities, possibly in order to facilitate further learning of regularities. This attentional bias can be both beneficial in allowing more learning to occur, and costly in terms of perhaps hindering learning of new information elsewhere.

## Prior Knowledge of Object Associations Dictates how new Information is Acquired

As both a consequence and a driver of the attentional selection process, prior knowledge of object associations can guide how new information is learned and created. Knowledge of object relationships can be acquired automatically and implicitly through statistical learning, which involves the extraction of reliable co-occurrences between individual objects over space and time (e.g., Fiser and Aslin, 2001; Turk-Browne et al., 2005). This ability is present in early infancy (Saffran et al., 1996; Fiser and Aslin, 2002; Kirkham et al., 2002; Wu et al., 2011), and perhaps even from birth (Teinonen et al., 2009). A notable consequence of statistical learning is the generation of the knowledge that certain objects co-occur, and such knowledge is often implicit (Baker et al., 2004; Turk-Browne et al., 2005; Wu et al., 2011, 2013). This learning process occurs incidentally to the task without conscious intent, and can guide the spatial allocation of attention in a spontaneous, implicit, and persistent manner (Zhao et al., 2013; Yu and Zhao, 2015; Zhao and Luo, 2017).

Recent studies have demonstrated that prior knowledge of how objects are related to each other generates new knowledge of associations (Mole and Zhao, 2016; Luo and Zhao, 2016; Zhao and Yu, 2016). In Luo and Zhao (2016), participants were first exposed to a sequence of colored circle pairs, in which one circle appeared before another in a fixed order. For example, in the AB pair, A appeared before B, and in the BC pair, B appeared before C, where A, B, and C were circles of different colors. After learning the color circle pairs, participants automatically inferred new color pairs AC even though they never appeared together before. Both the prior knowledge and the newly acquired knowledge were implicit, in that no participant was explicitly aware of the pairs. Moreover, after acquiring the prior knowledge of one pair at one categorical level, participants implicitly inferred the same association at the subordinate level and the superordinate level, even if the subordinate or superordinate objects were never presented before. For example, after learning a city pair New York-Vancouver, participants could implicitly infer the corresponding park pair Central Park-Stanley Park, and the corresponding country pair United States-Canada. These results suggest that prior knowledge automatically generates new knowledge of object associations through transitive relations, even outside of explicit awareness. This study with young adults builds on infant studies demonstrating that prior knowledge of older regularities biases learning of new regularities (Marcus et al., 2007; Quinn and Bhatt, 2009; Lew-Williams and Saffran, 2012). Lew-Williams and Saffran (2012) exposed infants to disyllabic or trisyllabic nonsense words, and then a new set of disyllabic or trisyllabic nonsense words. Listening times showed that infants were able to learn words only when the words were uniformly disyllabic or trisyllabic throughout the entire experiment. Previous exposure to disyllabic words impaired the ability to learn trisyllabic words, and vice versa. Thus, prior knowledge about word length produces expectations that facilitate processing of future word information.

## Use of Prior Knowledge Across the Lifespan

Most of the aforementioned studies on the influence of prior knowledge on attentional selection and information acquisition were conducted with young adults (18–30 years of age). Extending these investigations to other age groups across the lifespan, including infants and older adults, would provide a deeper understanding of how prior knowledge may have an increasingly impactful role in determining neural and behavioral outcomes with increased age and experience. One particularly challenging question in developmental psychology is how infants and children learn to engage in top-down goal-driven search to identify and learn about relevant events in the naturalistic, distraction-filled environment (Wu and Kirkham, 2010; Wu et al., 2011). Infants lack extensive prior knowledge due to their minimal exposure to the environment. Therefore, infants’ attention is initially driven by stimulus salience (e.g., luminance and high contrast) and biases, such as orienting toward the T configuration resembling faces (e.g., Johnson et al., 1991; Colombo, 2001). Infants rely heavily on external input (e.g., distributional statistics, Aslin and Newport, 2012) to search for information and learn about and from cues in the environment (e.g., social cues, Wu and Kirkham, 2010). For example, infants first learn about regularities of social cues such as direction of gaze, and then use this learned attentional cue to learn about objects by 8 months of age (Wu and Kirkham, 2010; Wu et al., 2014). On the other end of the lifespan, more research is required to investigate a new explanation that the seemingly worse cognitive performance in older adults may not accurately reflect actual cognitive decline, but rather the knowledge acquired over a lifetime (Ramscar et al., 2014; Blanco et al., 2016; Wu et al., 2017b). Ramscar et al. (2014) posit that increased general knowledge may induce retrieval issues that resemble memory decline because the learner has to sift through more prior knowledge, compared to younger age groups, to retrieve a specific piece of information. Wu et al. (2017b) argue that extensive prior knowledge may reduce broad learning experiences, which are prevalent during infancy and childhood. This reduction may encourage an increased reliance on prior knowledge, which may be a key factor driving the effects of apparent cognitive decline in healthy aging adults.

## Conclusion

In conclusion, studies that are based on the traditional view of attentional selection being neutral to semantic content (e.g., attending to a spatial location or using simple, meaningless search stimuli) have laid the foundation for the nature and limitations of attentional selection in specific simplified contexts. Recent studies have shown that prior knowledge of object associations influences attentional selection, determines the contents in attentional templates, and subsequently shapes information acquisition in beneficial and costly ways. This point is often underappreciated in research on visual search, as well as other aspects of cognition. Tasks that measure cognitive health across the lifespan typically “remove” the participant’s ability to use prior knowledge by using unpredictable meaningless stimuli. Given that prior knowledge can impact the efficiency in completing cognitive tasks, even when the knowledge is task-irrelevant, simple stimuli may underestimate or overestimate a participant’s abilities. These insights highlight the importance of the acquisition of appropriate prior knowledge and its use in cognitive tasks both in the laboratory setting, as well as in the natural environment. More research on how prior knowledge interacts with cognitive processes would lead to an increased emphasis on how the use of prior knowledge (e.g., for a search template) is optimized and how and when new information is acquired. Future research also could determine whether cognitive abilities should be conceptually separated from prior knowledge, for example as distinct layers of influence on performance, rather than inherently integrated (see Churchland et al., 1994). These efforts would lead to more accurate assessments of cognitive abilities and more effective training of these processes.

## Author Contributions

All authors listed have contributed equally to the work, and approved it for publication.

## Conflict of Interest Statement

The authors declare that the research was conducted in the absence of any commercial or financial relationships that could be construed as a potential conflict of interest.
